# Molecular cloning, characterization, and functional analysis of the uncharacterized *C11orf96* gene

**DOI:** 10.1186/s12917-022-03224-5

**Published:** 2022-05-10

**Authors:** Hongzao Yang, Jie Zhu, Hongyuan Guo, Aoxing Tang, Shaoyu Chen, Da Zhang, Ligang Yuan, Guangqing Liu

**Affiliations:** 1 College of Veterinary Medicine, Gansu Agricultural University, Lanzhou, 730070 People’s Republic of China; 2 Innovation Team of Small Animal Infectious Disease, Shanghai Veterinary Research Institute, Chinese Academy of Agricultural Sciences, Shanghai, 200241 People’s Republic of China

**Keywords:** C11orf96, *Felis catus*, Gene cloning, Expression patterns, Biological function

## Abstract

**Background:**

The mammalian genome encodes millions of proteins. Although many proteins have been discovered and identified, a large part of proteins encoded by genes are yet to be discovered or fully characterized. In the present study, we successfully identified a host protein C11orf96 that was significantly upregulated after viral infection.

**Results:**

First, we successfully cloned the coding sequence (CDS) region of the cat, human, and mouse C11orf96 gene. The CDS region of the C11orf96 gene is 372 bp long, encodes 124 amino acids, and is relatively conserved in different mammals. From bioinformatics analysis, we found that C11orf96 is rich in Ser and has multiple predicted phosphorylation sites. Moreover, protein interaction prediction analysis revealed that the protein is associated with several transmembrane family proteins and zinc finger proteins. Subsequently, we found that C11orf96 is strictly distributed in the cytoplasm. According to the tissue distribution characteristics, C11orf96 is distributed in all tissues and organs, with the highest expression levels in the kidney. These results indicate that C11orf96 may play a specific biological role in the kidney.

**Conclusions:**

Summarizing, these data lay the foundation for studying the biological functions of C11orf96 and for exploring its role in viral replication.

**Supplementary Information:**

The online version contains supplementary material available at 10.1186/s12917-022-03224-5.

## Background

Protein is known as the building block of life. Complicated life activities involve millions of proteins, which form an orderly life body through strict distribution and program control [1]. Currently, the proteins whose biological functions have been identified account for only a very small portion of the total proteins. Many proteins with unknown functions are yet to be discovered. In recent years, new viruses have emerged which pose a serious threat to humans as well as to livestock and poultry breeding. For example, severe acute respiratory syndrome coronavirus 2 (SARS-CoV-2) discovered in 2019 has been infecting hundreds of millions of people and has triggered a worldwide pandemic [2–4]. The African swine fever virus (ASFV) was introduced in China in 2018, and it has caused widespread death of pigs across the country; moreover, its high morbidity and mortality rate led to huge economic losses of the Chinese breeding industry [5]. Viruses are strictly parasitic organisms. They manipulate the translation system of the host and use raw materials in host cells to complete their self-replication and reproduction [6]. Therefore, exploring the mechanisms of interaction between a virus and its host and discovering the host proteins that regulate virus replication has always been a research hot spot in the field of life sciences.

With the advancement of science and technology, an increasing number of host proteins involved in regulating virus replication have been identified. Many cellular functional receptors of viruses have been discovered. For example, ACE2 is the receptor for human coronavirus, LDLR is the receptor for hepatitis C virus and rhinovirus, and CD46 is the receptor for classical swine fever virus, adenovirus, and human herpesvirus 6A [7–11]. Moreover, many host proteins involved in viral replication and translation have also been identified, such as eIF4E, eIF3, eIF4F, RPS5, RPS6, PABP, PTB, and VAPA/VAPB [12–17]. In addition, many host restriction factors with antiviral effects are expressed in viral infection, such as ZAP, ISG15, MX1, OAS, viperin, and tetherin [18–22]. Previously, we used the rabbit haemorrhagic disease virus (RHDV) as a model to perform proteomic analysis of viral infections and found that many host proteins were significantly upregulated after viral infection, including some uncharacterized proteins (data not shown). Proteomics data has been uploaded to ProteomeXchange database (Project accession: PXD030318). Among these unknown proteins, C11orf96 was found to be a host factor with the highest upregulation level. C11orf96 is a protein encoded by the 96th open reading frame on chromosome 11, and although its gene has been reported, the function of this protein remains to be identified [23]. Human chromosome 11 carries 1,524 protein-coding genes; although this chromosome is average in size, it is one of the most gene- and disease-rich chromosomes in the human genome. For example, modifications of nuclear DNA and its regulatory proteins (IGF2, SLC22A18, PHLDA2, CDKN1C, and KCNQ1) and proteins involved in cancer development (MYCN, IGSF4, and CADM1) are known to be located on human chromosome 11 [24–27]. However, the functions of only a part of the proteins encoded on chromosome 11 have been elucidated.

In the present study, we successfully cloned the C11orf96 gene and analyzed its potential functions by using bioinformatics tools. Quantitative PCR, western blotting (WB) assay, immunofluorescence assay (IFA), and immunohistochemistry were used to analyze the distribution of the C11orf96 gene in cells and tissues. The obtained data could provide important clues for studying the biological functions of the C11orf96 protein.

## Results

### Cloning and expression of the C11orf96 protein

**Fig. 1:**
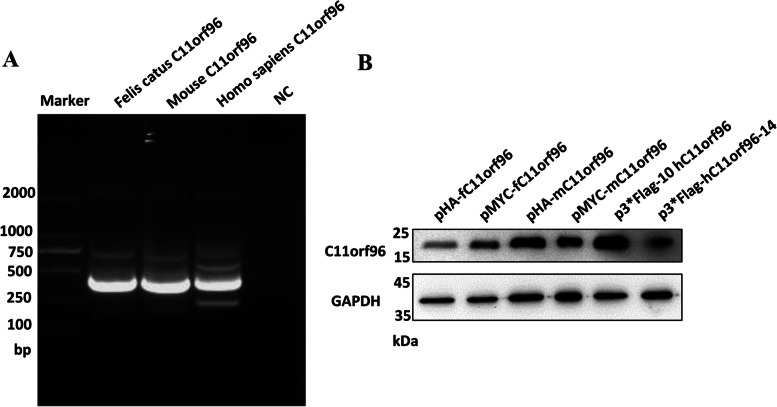
Gene cloning and protein expression of the *C11orf96* gene from different sources**. A** PCR amplification derived from different cDNAs of *Felis catus*, mouse, and *Homo sapiens*. M, DL2000 marker. **B** Western blotting. The antibodies prepared in our own laboratory were used to express different gene proteins

The *C11orf96* gene in the bait vector pEASY®-Blunt Zero Cloning Kit (abbreviated as pEBCK) was obtained by RT-PCR, and 1% agarose gel electrophoresis showed that the size of the cDNA fragment amplified by PCR was in the expected range. The target fragment size of 372 bp (Fig. 1A) was confirmed by sequencing analysis. Sequencing analysis also confirmed that the insert was the C11orf96 CDS, indicating successful construction of the bait vector pEBZCK–Felis catus C11orf96, pEBZCK-mouse C11orf96, and pEBZCK-homo sapiens C11orf96. Blast search and comparison with the NCBI nucleotide sequence database revealed that the CDS was completely consistent with the *F. catus* C11orf96 CDS region sequence (XM_006937308.4), mouse C11orf96 (NM_001145034.1), and *H. sapiens* C11orf96 (NM_001145033.2) used in the design of the cloning primer. Moreover, we successfully constructed eukaryotic expression plasmids of C11orf96 (pHA-fC11orf96, pMYC-fC11orf96, pHA-mC11orf96, pMYC-mC11orf96, pFlag-hC11orf96, and phC11orf96-Flag). After these eukaryotic plasmids were transfected into 293 T cells, the cell lysate was collected for WB assay. The results showed that *F. catus*, mouse, and *H. sapiens* C11orf96 eukaryotic plasmids were effectively expressed (Fig. 1B).

### Analysis of biological characteristics of C11orf96

**Fig. 2:**
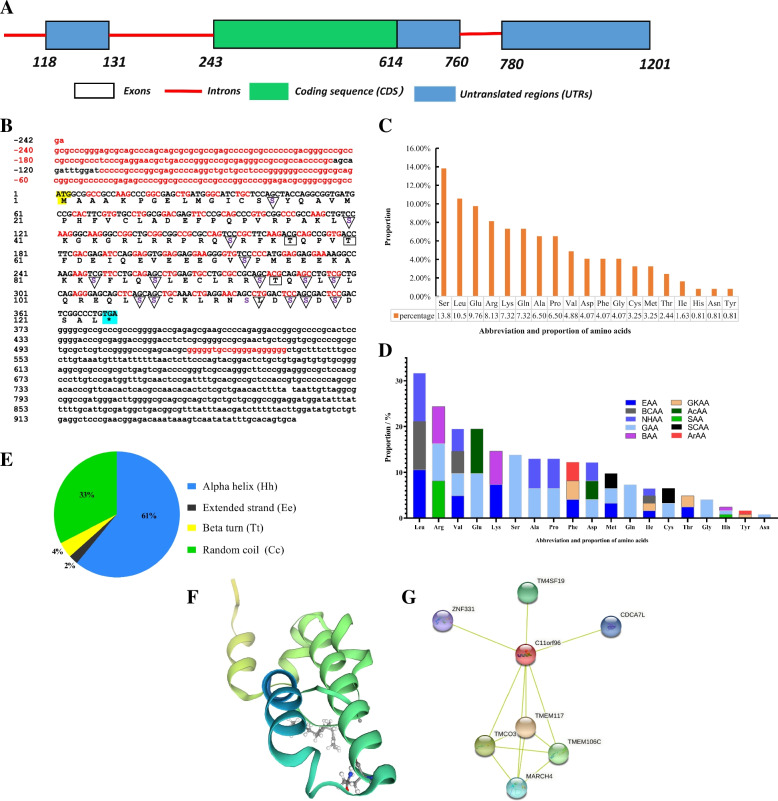
Analyses of biological characteristics of C11orf96. **A** Structural diagram of the *Felis catus C11orf96* gene (sequence data are available from GenBank: accession No. XM_006937308.4). **B** The nucleotide and deduced amino acid sequences for the cloned C11orf96 CDS region. ATG and TAA are the start and stop codons, respectively. Serine phosphorylation sites are marked with ▽. Threonine phosphorylation sites are marked with ▭. **C** Diagram showing the percentages of the amino acids in *F. catus* C11orf96. **D** Classification of amino acids in C11orf96 according to their nature. **E–F** Pie chart shows the C11orf96 secondary structure composition and rertiary structure. **G** Schematic diagram of the network of tight proteins interacting with the C11orf96 protein. Note: EAA, essential amino acid; SAA, semi-essential amino acid; GAA, glycogenic amino acid; GKAA, glucogenic and ketogenic amino acid; BAA, basic amino acid; AcAA, acidic amino acid; BCAA, branched chain amino acid; NHAA, nonpolar hydrophobic amino acid; ArAA, aromatic amino acid; SCAA, sulfur-containing amino acid

The full length of *F. catus* C11orf96 is 1201 bp, with 3 introns and 3 exons. The untranslated regions (UTRs) are located at 118–131, 615–760, and 780–1201 bp; the CDS region is located at 243–614 bp and is 372 bp long (Fig. 2A). The cloned sequence has 8 ORFs, among which the full-length open reading frame ORF1 (the complete CDS region) is 372 bp, which encodes 124 amino acids, including predicted phosphorylation sites (Tyr: 3, Ser: 15). The protein sequence does not contain a signal peptide and does not have a transmembrane region (Fig. 2B). The top five amino acids are Ser (13.82%) > Leu (10.57%) > Glu (9.76%) > Arg (8.13%) > Lys (7.32%). The detailed amino acid composition ratio is shown in Figs. 2C and D. The molecular weight is 13.80 kDa, the isoelectric point (pI) is 8.4, and the molecular formula is C_592_H_970_N_174_O_189_S_8_. The detailed physical and chemical properties are shown in Table S1. The protein secondary structure prediction revealed that the C11orf96 protein consists of four structures: α-helix, β-turn, random coil, and extended chain, which account for 61%, 4%, 33%, and 2% of the protein structure, respectively (Fig. 2E-F). Protein interaction prediction analysis showed that the C11orf96 protein may interact with multiple proteins in the host, including the TMEM117 transmembrane protein that regulates endoplasmic reticulum (ER) stress, several other transmembrane proteins, E3 ubiquitin ligase, and zinc finger proteins (Fig. 2G). These results indicate that the C11orf96 protein may play a role in cellular processes such as ER stress, protein ubiquitination modification, and gene transcription.

### Conservation analysis of C11orf96 in different species

**Fig. 3:**
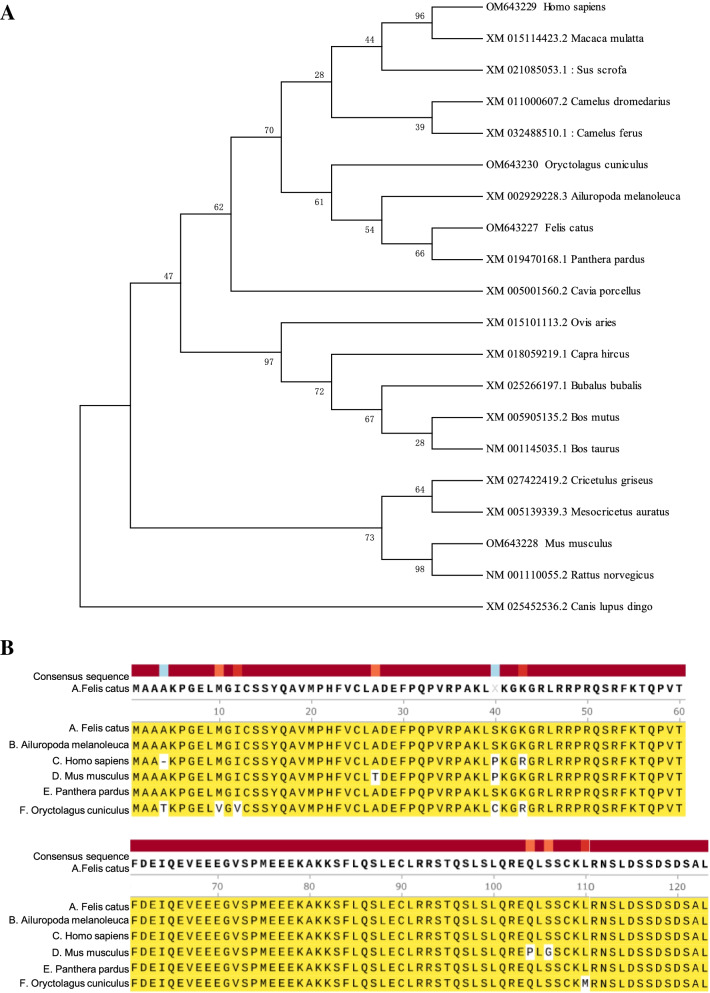
Conservation analysis of C11orf96 in different species. **A** Phylogenetic tree for amino acid sequences of the C11orf96 protein based on the neighbor-joining method. The phylogenetic tree is generated by MEGA7.0 using the neighbor-joining method with 1000 bootstrap replicate. GenBank accession numbers for the sequence are shown in Table S2. The bootstrap values and branch lengths are shown above and below each branch, respectively. A closer phylogenetic relationship with *Felis catus* C11orf96 is indicated by the asterisk. **B** Alignment of the deduced amino acid sequences of *F. catus* with those of other mammals. The same amino acids and conserved amino acids of different species are expressed in the same color

The phylogenetic tree was used to analyze the amino acid sequence of C11orf96 from 20 species of mammals. It was found that the amino acid sequences of C11orf96 of *F. catus* and *Panthera pardus* are clustered together and are evolutionarily closely related to those of *Ailuropoda melanoleuca*, *Ovis aries*, *Capra hircus*, and *Bubalus bubalis* (Fig. 3A), which is consistent with the results of amino acid sequence homology comparison. These results indicate that the C11orf96 protein is relatively conserved in different species such as rabbits, cats, mice, and humans, and the major difference lies in the N terminal sequence. Sequence alignment revealed that the 124-amino-acid mature peptides of C11orf96 are well conserved in different mammals (Fig. 3B). Comparison with *F. catus* amino acids showed that C11orf96 is highly conserved between *P. pardus* and *A. melanoleuca* (100% identity), and the only difference when compared with *Mus musculus* (96.0% identity) was noted in amino acids at 4 sites: 27 (Thr), 40 (Pro), 104 (Pro), and 106 (Gly). The amino acid sequence also showed homology with those of *Oryctolagus cuniculus* (94.3% identity) and *H. sapiens* (95.9% identity). These results indicate that C11orf96 is conserved in mammals.

### The C11orf96 protein is localized in the cytoplasm

**Fig. 4:**
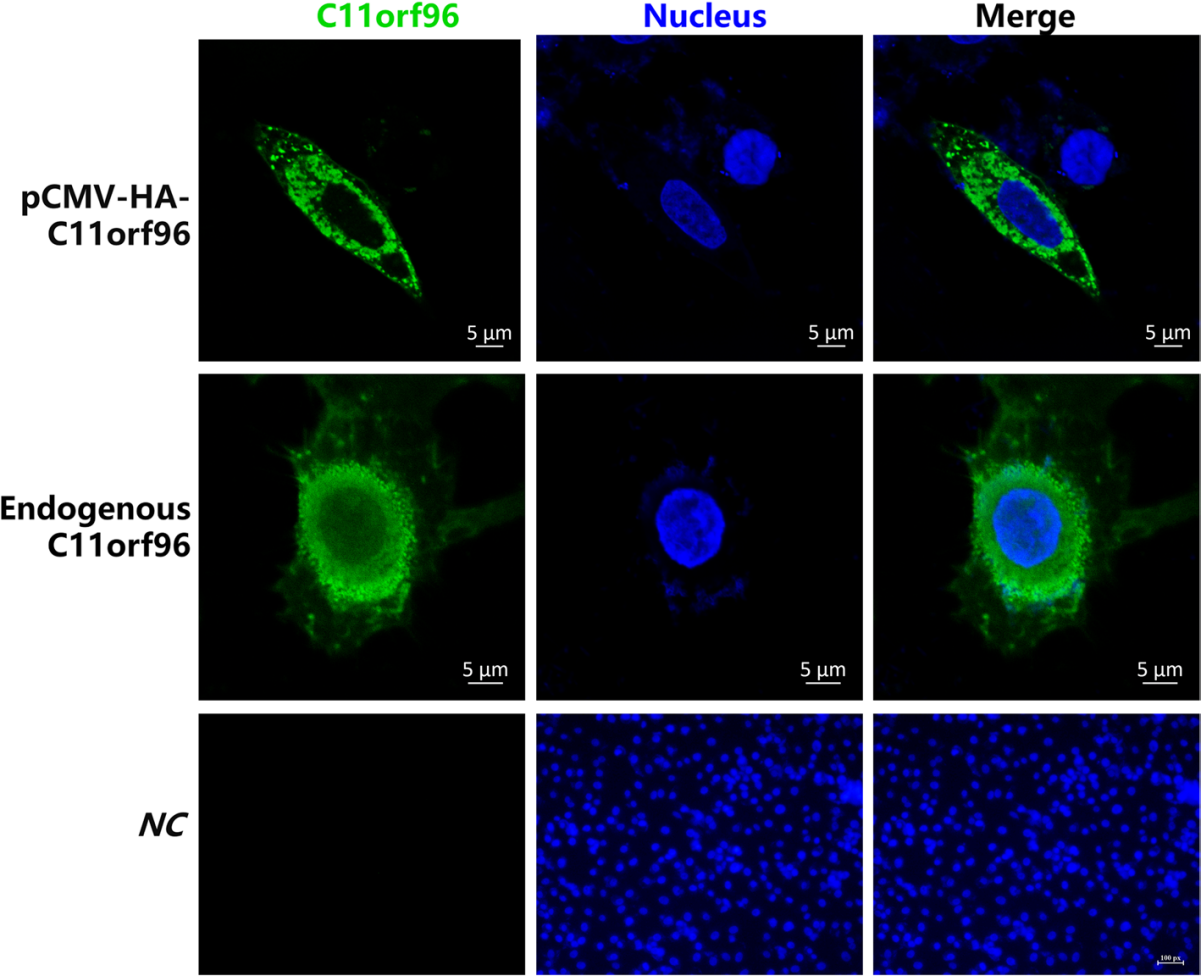
Subcellular localization of the C11orf96 protein. Left panels, transient expression of pCMV-HA-tagged C11orf96 and endogenous C11orf96 in CRFK cells visualized by epifluorescence microscopy; middle panels, DAPI staining to visualize nuclei; right panels, merged images. Bars = 20 μm

The IFA experiment with the HA tag antibody was performed to analyze the distribution of exogenous HA-c11orf96 in CRFK cells. As shown in Fig. 4, HA-C11orf96 was expressed only in the cytoplasm. We also used the C11orf96 polyclonal antibody prepared in our laboratory to detect the expression of endogenous C11orf96 in CRFK cells. The result was similar to that observed for exogenous HA-C11orf96, that is, endogenous C11orf96 was expressed only in the cytoplasm (Fig. 4). These data clarify the distribution of C11orf96 in cells.

### C11orf96 is mainly distributed in kidney

**Fig. 5:**
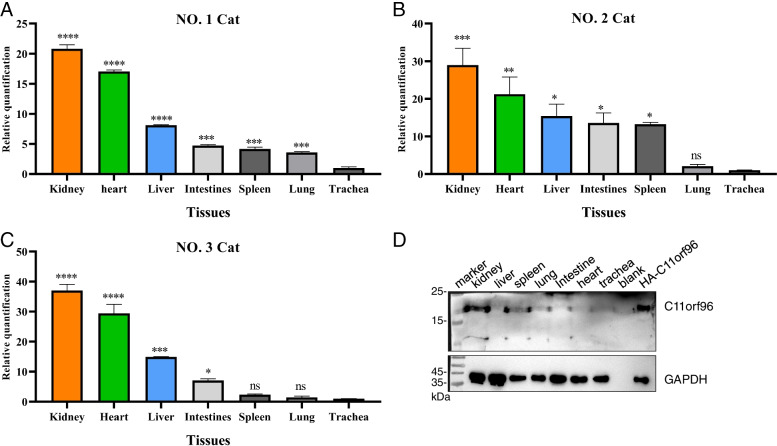
The mRNA and protein expression levels of *Felis catus* C11orf96 in different tissues. **A-C** The dynamic range of qPCR for C11orf96 quantification in the kidney, liver, spleen, lung, intestine, heart, and trachea. The trachea was used as a reference for data analysis. The β-actin acted as an internal reference gene. The trachea served as the analytical reference. All data are obtained from three independent experiments. **D** The protein expression level in different tissues was determined by western blotting assay. GAPDH was used as the housekeeping gene control. Rabbit polyclonal antibody for C11orf96 was prepared in our laboratory

**Fig. 6:**
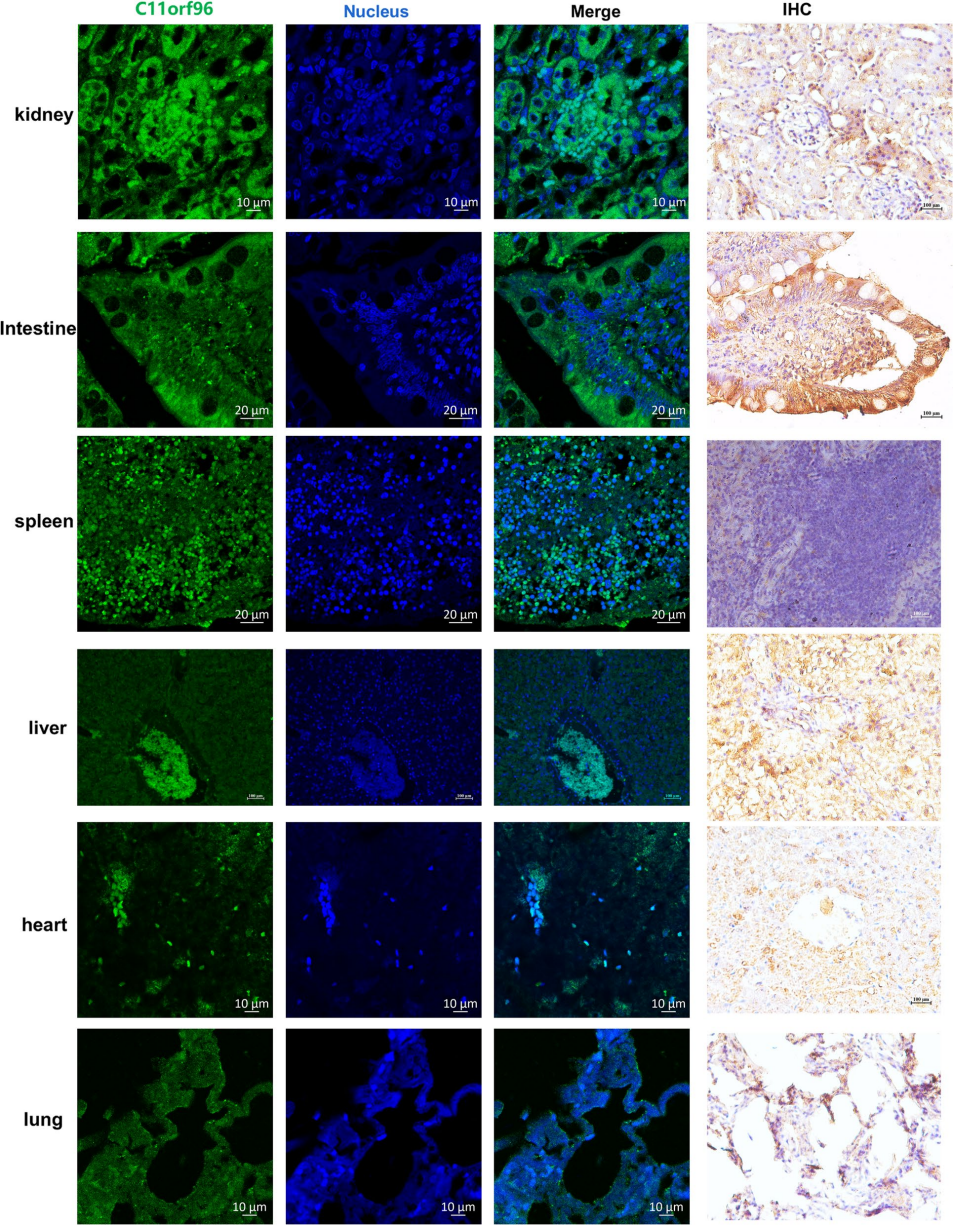
Immunofluorescence and immunohistochemical staining of the C11orf96 protein in different tissues. Green tissues indicate fluorescence coloration due to FITC-conjugated or Alexa Fluor 488-conjugated secondary antibodies. Black area indicates background. Blue tissue indicates fluorescence coloration of nuclear labeling by DAPI

Relative quantitative PCR was used to analyze the transcription level of C11orf96 in different tissues. As shown in Fig. 5A, C11orf96 showed the highest mRNA transcription levels in the kidney. Subsequently, we analyzed the distribution of C11ORF96 in two other cat tissues, and found that C11orf96 also showed the highest transcription level in kidney, and the expression trend was similar in all cats (Fig. 5B-C). Moreover, we extracted samples of total protein from each tissue and performed WB assay with the C11orf96 antibody. The results showed that the protein was highly expressed in the kidney (Fig. 5D). In addition, immunohistochemistry and IFA were performed with the C11orf96 antibody. As shown in Fig. 6, C11orf96 exhibited the highest expression level in kidney. We performed WB and immunohistochemical analysis of tissue from all three cats and obtained results similar to those presented above. It is worth noting that the transcription level of C11orf96 in the heart is high, but the protein level is low, indicating that the C11orf96 protein may be degraded in the heart, and its mechanism needs to be further studied. Summarizing, these results indicate that C11orf96 is mainly distributed in the kidney in normal tissues, suggesting that C11orf96 may be involved in the biological activities of the kidney.

## Discussions

C11orf96 is encoded by the 96th ORF on chromosome 11, and its biological characteristics and functions are unclear. Chromosome 11 is a pair of chromosomes containing the most genes for disease control in the human genome, such as IGF2, SLC22A18, CDKN1C, MYCN, IGSF4, and CADM1 [28–30]. C11orf96 is a protein that is significantly upregulated after virus infection. Therefore, understanding the biological characteristics of the C11orf96 protein is critical to study its biological functions in viral infections.

The protein functional domain prediction analysis showed that C11orf96 has no transmembrane structure and no signal peptide and consists of some low complexity regions, a functional domain DUF4695 of 109–206 amino acids with unclear function, and a highly conserved RFKTQP motif. From the Interpro domain database, we found that DUF4695 is usually associated with Alpha-ketoglutarate-dependent dioxygenase AlkB-like (AlkB-like), Short-chain dehydrogenase/reductase SDR (SDR_fam), Pleckstrin homology domain (PH_domain), and Integrase, core catalytic (Integrase_cat) domains appear together. AlkB is a DNA repair enzyme that removes methyl adducts and some larger alkylation lesions from endocyclic positions on purine and pyrimidine bases [31]. SDR_fam is a very large family of enzymes, most of which are known to be NAD- or NADP-dependent oxidoreductases [32]. PH domains can bind to phosphatidylinositol in biological membranes and proteins such as the beta/gamma subunits of heterotrimeric G proteins and protein kinase C [33]. Through these interactions, PH domains play a role in recruiting proteins to different membranes, thus targeting them to appropriate cellular compartments or enabling them to interact with other components of the signal transduction pathways [34, 35]. The integrase catalytic domain catalyzes a series of reactions to integrate the viral genome into a host chromosome [36]. Therefore, it can be speculated that the DUF4695 domain may regulate the activity of the above functional domains and that C11orf96 plays a regulatory role in these biological activities.

The proportion of serine in the C11orf96 protein is as high as 13.82%, and the protein was predicted to contain 15 potential serine phosphorylation sites. Protein phosphorylation is one of the most important post-translational modifications of proteins, which regulates almost all cellular activities [37]. Protein phosphorylation is the transfer of ATP phosphate to the amino acid residues of the substrate protein under the catalysis of protein kinase and the main phosphorylated residues are serine, threonine, and tyrosine. Phosphorylation is an important post-translational modification in signal transduction and is related to many protein interaction events [38, 39]. Studies have shown that the phosphorylation of host cell kinase is closely related to virus replication and gene expression [40]. Proteins always perform their biological functions by interacting with other proteins. By using the protein interaction prediction database STRING, we found that C11orf96 may interact with proteins such as ZNF331, TM4SF19, CDCA7L, MARCH4, TMCO3, TMEM106C, and TMEM117. MARCH4 is a E3 ubiquitin-protein ligase that is predicted to mediate ubiquitination of MHC-I and CD4 and promote their subsequent endocytosis and sorting to lysosomes through multivesicular bodies [41]. CDCA7L plays a role in transcriptional regulation as a repressor that inhibits monoamine oxidase A (MAOA) activity and gene expression by binding to the promoter and is involved in apoptotic signaling pathways [42, 43]. TMEM117 is involved in ER stress-induced cell death pathway [44]. ZNF331 may be involved in transcriptional regulation [45]. These results indicate that C11orf96 may use phosphorylation to play a role in ER stress, protein ubiquitination modification, gene transcription, and other cellular processes.

The analysis of the distribution of C11orf96 in each tissue showed that the expression levels of C11orf96 were the highest in the kidney. C11orf96 was mainly concentrated in glomerular epithelial cells. Therefore, we presumed that C11orf96 plays a role in the formation of renal tubules during kidney development. In addition, C11orf96 was also expressed in the spleen, suggesting that this gene may be involved in some biological activities in the spleen. The spleen is the most important immune organ and the main place to resist foreign pathogens by providing humoral immunity and natural immune response [46]. C11orf96 is widely distributed in the spleen, indicating that this protein may be involved in the body’s defense against foreign pathogens. As observed earlier, the expression level of C11orf96 was significantly upregulated after viral infection, indicating that this protein is involved in regulating the life cycle of the virus. In the future, we plan to investigate the molecular mechanisms through which C11orf96 regulates viral replication as the research direction to conduct further studies on its specific biological functions.

## Conclusions

In this study, the uncharacterized C11orf96 gene that is conserved in mammals was successfully cloned. We found that this protein is expressed only in the cytoplasm. We also found that C11orf96 is expressed at higher levels in the kidney. These findings lay important foundation for studying the specific biological functions of C11orf96.

## Methods

### Tissue collection

Tissues were collected from three healthy stray cats from a pet hospital in Shanghai, China. On the day of necropsy, cats were initially sedated and then euthanized by intravenous injection of 85.9 mg/kg pentobarbital sodium. The tissues of major organs such as heart, liver, spleen, lung, kidney, and intestine were obtained by ourselves immediately after euthanasia. Samples for gene cloning and real-time PCR were immediately placed in liquid nitrogen (-196℃), transported to the laboratory, and stored at -80℃.The other part of the tissue sample was fixed in 4% paraformaldehyde solution for preparing paraffin sections. All experiments were performed according to the guidelines established by Shanghai Veterinary Research Institute, CAAS, China (approval number: SHVRIAU-18–035). All experiments were designed to minimize the number of animals used. All methods are reported in accordance with ARRIVE guidelines (https://arriveguidelines.org) for the reporting of animal experiments.

### Plasmids, antibodies, and cells

The C11orf96 gene was cloned into the pCMV-HA/MYC vector and the p3*Flag-10/14 vector (Clontech) by using the Clon Express Ultra One Step Cloning Kit (Vazyme, China) to obtain the pHA-Felis catus C11orf96 plasmid, pMYC-Felis catus C11orf96 plasmid, pHA-mouse C11orf96 plasmid, pMYC-mouse C11orf96 plasmid, p3*Flag-10-Homo sapiens C11orf96 plasmid, and p3*Flag-14-Homo sapiens C11orf96 plasmid. The specific plasmid construction methods are described in Sect. 2.3.

The antibodies used in this study included rabbit C11orf96 polyclonal antibody (prepared by our laboratory), mouse anti-Flag antibody (Sigma Aldrich), mouse anti-GAPDH antibody (Kangwei Century Biotechnology, China), goat anti-mouse IgG conjugated with HRP (Jackson ImmunoResearch Europe Ltd., USA), goat anti-rabbit IgG conjugated with HRP (Jackson ImmunoResearch Europe Ltd.), and goat anti-mouse IgG conjugated with Alexa Fluor 488 (Thermo Fisher Scientific, USA).

293 T cells and CRFK cells were cultured at 37℃ in a humidified incubator with 5% CO_2_ using Dulbecco’s modified Eagle’s medium (DMEM) and Eagle’s Minimal Essential Medium (Life Technologies, USA) containing 10% fetal bovine serum (FBS) (Biological Industries, Israel).

### Molecular cloning of the C11orf96 cDNA and construction of eukaryotic expression plasmids

For cDNA cloning, we used Trizol reagent to extract total RNA from frozen cat tissues, feline kidney cells (CRFK) cells, 293 T cells, and RAW 264.7 cells. The cDNA was then generated using M-MLV reverse transcriptase and random primers (Promega, USA). According to the *Felis catus C11orf96* (XM_006937308.4), mouse *C11orf96* (NM_001145034.1), and *Homo sapiens C11orf96* (NM_001145033.2), we designed amplification primers for the coding region of the *C11orf96* gene (Table 1). These primers were synthesized by GENEWIZ (Suzhou, China). By using the cDNA obtained by reverse transcription as the template, the CDS region of the *C11orf96* gene of *F. catus*, mouse, and *H. sapiens* was amplified by RT-PCR. The PCR products were then separated by 1% agarose gel electrophoresis. The target product was purified and recovered by a gel recovery kit (Vazyme, China), linked to the pEASY®-Blunt Zero Cloning vector (TransGen Biotech, China), and sequenced by Shanghai Sonny Biotech Co., Ltd. (China).

**Table 1:** The list of primer information in this study

Primers	Sequence (5′-3′)	Application
cat-C11orf96-F	ACCCCGCAGCAGATTTGGATC	Cat C11orf96 clone
cat-C11orf96-R	AGAGTGTGTTGGCGTGAGTGT	
mouse-C11orf96-F	AGAGGCGGGCTATATAAGCGGCTA	Mouse C11orf96 clone
mouse-C11orf96-R	GTGTTCAGCGAAAGTGTCGGC	
Homo sapiens-C11orf96-F	CCACCCCGCAGCAGATTTGGA	Homo sapiens C11orf96 clone
Homo sapiens-C11orf96-R	TCCCCGCACACACTCACAGCA	
cC11orf96-myc/HA-F	ATGGAGGCCCGAATTCGGATGGCGGCCGCCAAGCCCGGCGAGCTG	Cat C11orf96 clone in pCMV-Myc/HA vector
cC11orf96-myc/HA-R	GTACCTCGAGAGATCTTTACAGGGCCGAGTCGGAGTCGCT	
mC11orf96-myc/HA-F	ATGGAGGCCCGAATTCGGATGGCGGCCGCCAAGCCCGGCGAGCTC	Mouse C11orf96 clone in pCMV-Myc/HA vector
mC11orf96-myc/HA-R	GTACCTCGAGAGATCTTTACAGGGCCGAGTCGGAGTCGCT	
hC11orf96-p3*Flag10/14-F	ATTCATCGATAGATCTGATGGCCGCCAAGCCCGGCGAGCTG	Homo sapiens C11orf96 clone in pCMV-Flag-10/14 vector
hC11orf96-p3*Flag10/14-R	AGAGTCGACTGGTACCGATTACAGGGCCGAGTCGGAGTCGCT	
cC11orf96-qF	GTGACCTTCGACGAGATCCAGGAG	Measurement of cat C11orf96
cC11orf96-qR	GAGTCGGAGTCGCTGGAGTCC	
cβ-actin-qF	CTGGTATTGTCATGGACTCTG	Measurement of cat β-actin
cβ-actin-qF	CTCCAGGGAGGACGAGGAC	

### Bioinformatics and phylogenetic analyses

The sequences of the cloned *C11orf96* gene were confirmed using the BLAST tool available at the National Center for Biotechnology Information (NCBI) website. The genetic relationship and sequence similarity, and the amino acid sequence homology of C11orf96 of different species were compared with DNAMAN 8.0 software. The ORF Finder online tool in NCBI was used to search and analyze all open reading frames (ORFs) in the C11orf96 CDS region. The physical and chemical properties, functional domain, secondary structure, and tertiary structure of the C11orf96 protein were analyzed by the online software ProtParam, InterPro, SOPMA, and Phyre 2.0, respectively. The transmembrane region and the signal peptide region of this protein were predicted by TMHMM 2.0 and the SignalP software, respectively. A phylogenetic tree of C11orf96 was constructed by MEGA 7.0 software. The STRING 11.0 database was used for the analysis of potential proteins interacting with the C11orf96 protein, and the results were visualized using Cytoscape 3.8.0 software.

### Subcellular localization

CRFK cells were seeded onto 12-well plates and then transfected or not transfected with the plasmid pCMV-HA-Felis catus C11orf96. The cells were then cultured for 24 h. Next, the cells were washed with cold PBS and fixed in 4% paraformaldehyde solution for 30 min at room temperature. The cells were then permeabilized with methanol for 10 min at -20℃and blocked with 5% bovine serum albumin (BSA) and 0.3% TritonX-100 in PBS for 2 h at room temperature. Subsequently, the cells were stained with primary antibodies (antibody HA, 1:1000; polyclonal antibody C11orf96, 1:100) overnight at 4℃ and then with secondary antibodies (Alexa Fluor 488-conjugated goat anti-rabbit IgG (H + L), 1:1000) in a blocking buffer of 5% BSA in PBS for approximately 1–2 h at room temperature in dark. The cells were washed in PBS after each incubation with antibodies and then stained with 4ʹ,6-diamidino-2-phenylindole (DAPI, Thermo Fisher Scientific) for approximately 4–5 min and then washed four times with PBS. The images were acquired with a Zeiss LSM880 confocal microscope and analyzed by Zen Blue software (Zeiss, Germany).

### Analysis of expression patterns of *F. catus* C11orf96

The transcription levels of the *C11orf96* gene in each tissue were determined using the relative quantification of gene transcripts and the β-actin gene as an internal control. The cDNA samples were subjected to real-time PCR with SYBR Green Pro Taq HS Premix (Accurate Biology, Hunan, China) using an ABI 7500 Fast Real-Time PCR system (Applied Biosystems, USA). The primers are listed in Table 1. The data were calculated with the 2^−△△CT^ method and the transcription levels of the *C11orf96* gene was analyzed by GraphPad Prism 8.0. Subsequently, immunohistochemistry and IFA were used to analyze the expression of the *C11orf96* gene in each tissue. The tissue samples fixed in 4% paraformaldehyde were embedded in paraffin, and 4-μm-thick paraffin sections were prepared. Rabbit C11orf96 polyclonal antibodies (dilution ratio 1:100, prepared by our laboratory) were used to perform immunohistochemistry and IFA experiments in accordance with routine protocols. HRP-labeled goat anti-rabbit IgG secondary antibodies and goat anti-rabbit FITC secondary antibodies were used in these assays. The images were acquired with a Zeiss LSM880 confocal microscope and analyzed by Zen Blue software (Zeiss, Germany).

### WB assay

Protein samples were separated on 12% gels and then transferred to nitrocellulose membranes (Hybond-C; Amersham Life Sciences, UK) by using a semi-dry transfer apparatus (Bio-Rad Laboratories, USA). The membranes were blocked with 5% (w/v) nonfat milk in TBST buffer (150 mM NaCl, 20 mM Tris, and 0.1% Tween-20; pH 7.6) for 3 h at 4 °C and then stained overnight at 4 °C with rabbit polyclonal C11orf96 antibodies (1:350) or GAPDH antibodies. After washing the membrane three times with TBST (10 min/time), goat anti-rabbit IgG secondary antibodies (1:10,000) were added for 1 h at room temperature. The membrane was then cleaned three times with TBST (10 min/time) at room temperature. The bands were detected by the enhanced chemiluminescence kit (Thermo Fisher Scientific, USA) by using the ECL luminescence solution for chemiluminescence, exposure, and development.

### Statistical analyses

Data were analyzed by the statistical analysis software GraphPad Prism 8.0. Student’s t-test and analysis of variance were used for statistical analyses. *p < 0.05, **p < 0.01, ***p < 0.001 and ****p < 0.0001 were considered to be statistically significant.

## Supplementary Information

**Additional file 1:** **Fig S1.** and **Fig S5.** Uncropped images.

**Additional file 2:** **Table S1.** The detailed physical and chemical properties of C11orf96.

**Additional file 3:** **Table S2.** The species of C11orf96 gene in this study.

## Data Availability

The datasets and materials used and/or analyzed during the current study are available from the corresponding author on reasonable request. The coding RNA sequences of the *Felis catus*, *Mus musculus*, and *Homo sapiens* C11orf96 genes, obtained in this study, have been uploaded to the genbank database GenBank accession numbers for these nucleotide sequences: *Felis catus* C11orf96 (OM643227), *Mus musculus* C11orf96 (OM643228) and *Homo sapiens* C11orf96 (OM643229).

## Abbreviations

CDS: Coding sequence
GAPDH: Glyceraldehyde 3- phosphate dehydrogenase
SARS-CoV-2: Severe acute respiratory syndrome coronavirus 2
ASFV: African swine fever virus
RHDV: Rabbit haemorrhagic disease virus
PCR: Polymerase Chain Reaction
WB: Western blotting
IFA: Immunofluorescence assay
UTRs: Untranslated regions; pI: isoelectric point
CRFK: Crandell-Rees Feline Kidney
ER: Endoplasmic reticulum
DMEM: Dulbecco’s modified Eagle’s medium
FBS: Fetal bovine serum
RT-PCR: Reverse transcription-polymerase chain reaction
ORF: Open reading frames
BSA: Bovine serum albumin
