# Supplementary material for: Molecular cloning, characterization, and functional analysis of the uncharacterized C11orf96 gene
Source: BMC Vet Res. 2022 May 10;18:170. doi: 10.1186/s12917-022-03224-5 (PMC9086667; doi:10.1186/s12917-022-03224-5)

Supplemental Fig 1 and Fig 5 Uncropped images

Figure 1A Uncropped PCR images

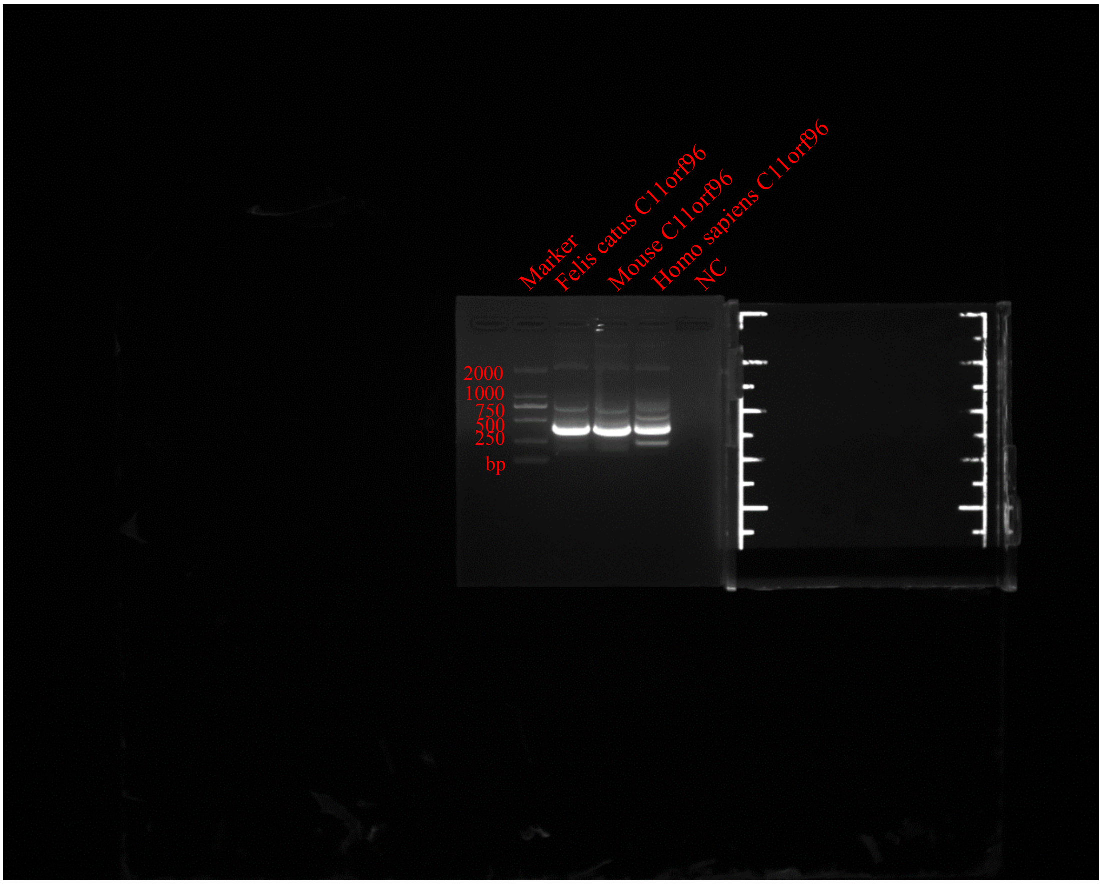

Figure 1B Uncropped WB images

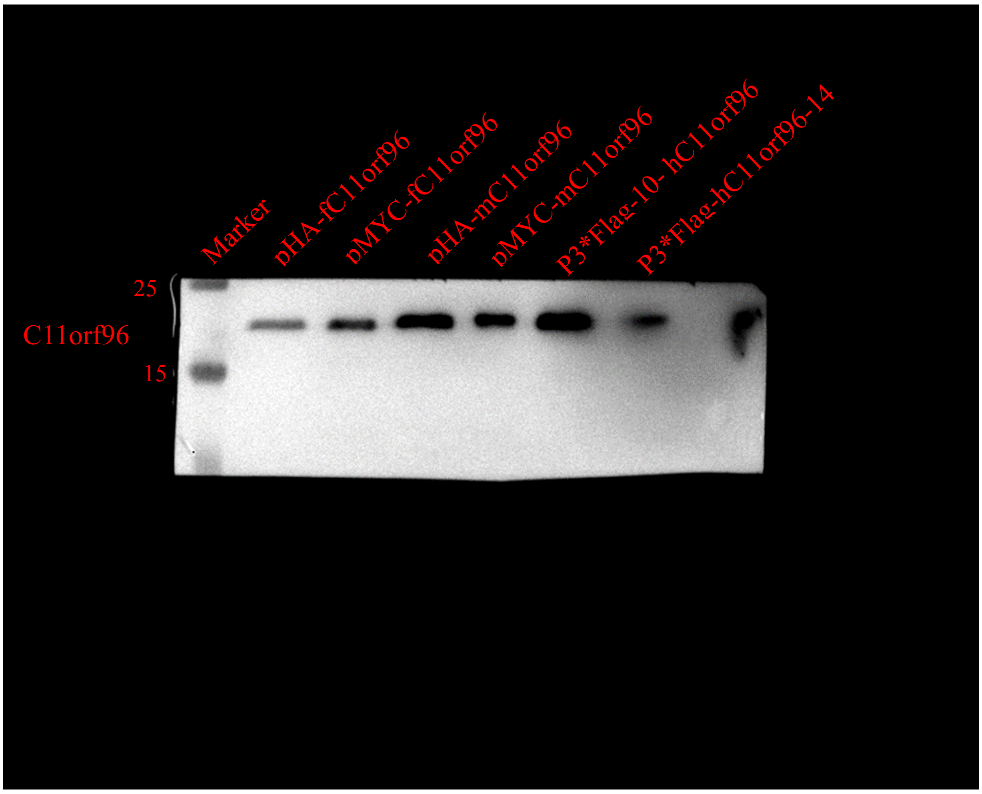

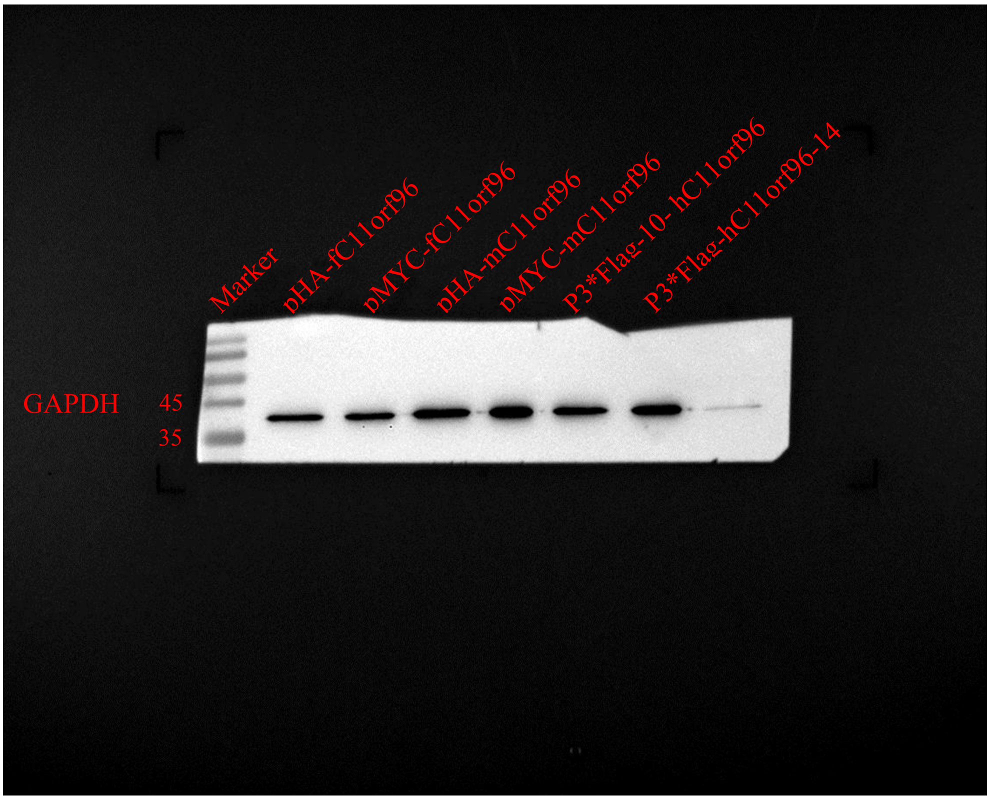

Figure 5D Uncropped WB images

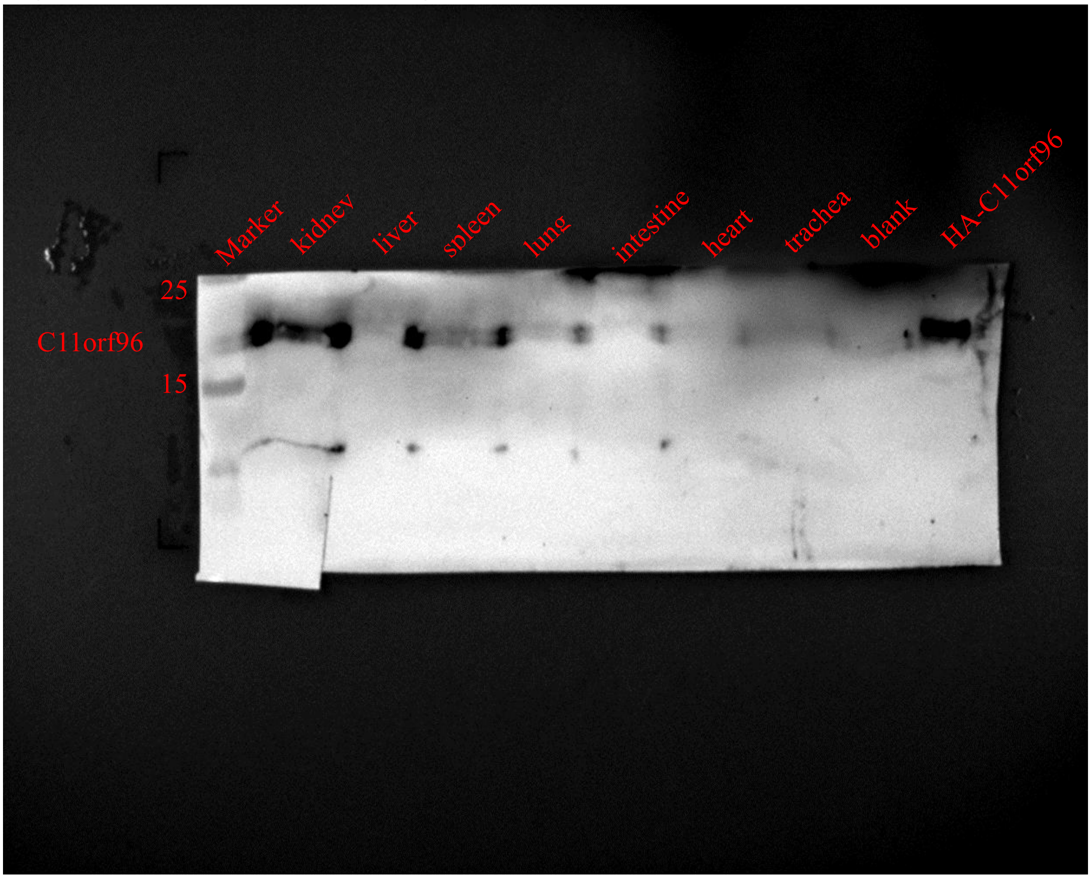

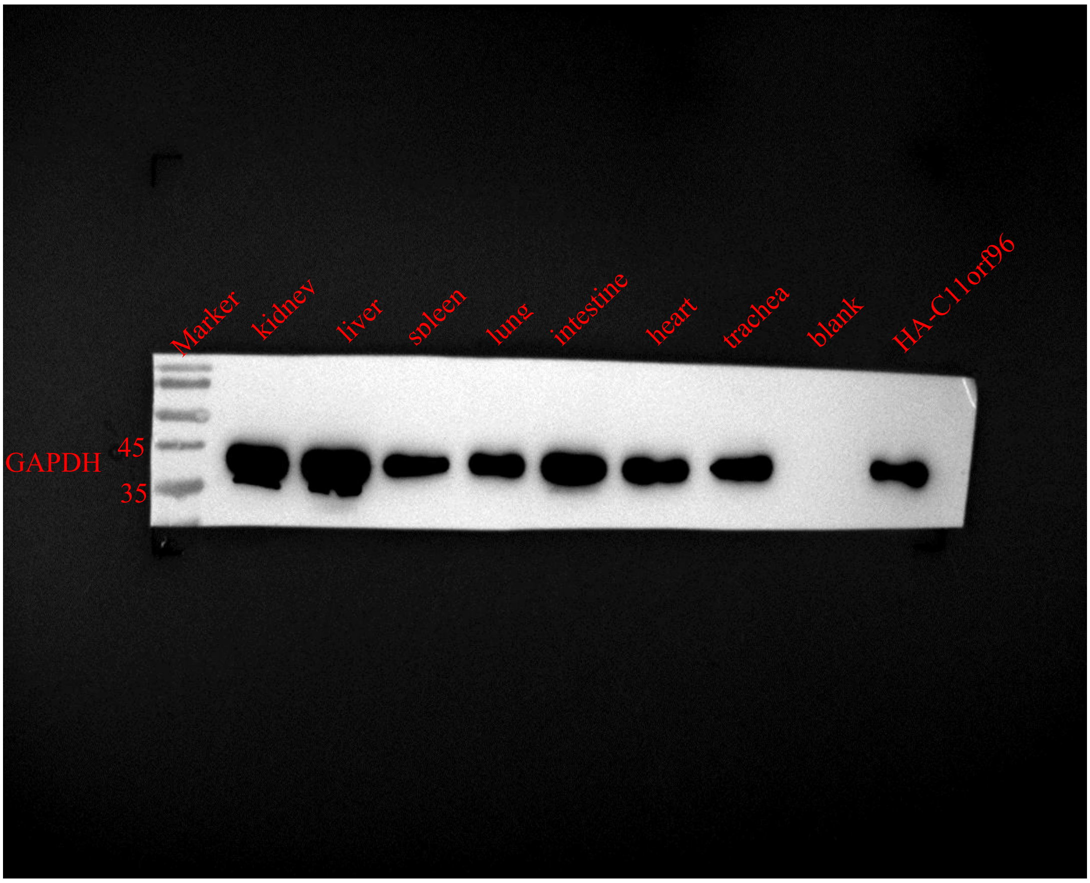

Supplement: Supplementary file 1 — Additional file 1: Fig S1. and Fig S5. Uncropped images. [file 12917_2022_3224_MOESM1_ESM.pdf]
