# Supplementary material for: Molecular cloning, characterization, and functional analysis of the uncharacterized C11orf96 gene
Source: BMC Vet Res. 2022 May 10;18:170. doi: 10.1186/s12917-022-03224-5 (PMC9086667; doi:10.1186/s12917-022-03224-5)
Supplement: Supplementary file 2 — Additional file 2: Table S1. The detailed physical and chemical properties of C11orf96. [file 12917_2022_3224_MOESM2_ESM.docx]

**Supplementary Table S1. The detailed physical and chemical properties of C11orf96.**

| **Physicochemical property** | **Prediction result** |
| --- | --- |
| Formula | C_592_H_970_N_174_O_189_S_8_ |
| Number of amino acids | 123 |
| Molecular weight | 13805.75 |
| Theoretical pI | 8.4 |
| Total number of negatively charged residues(Asp+Glu) | 17（13.83%） |
| Total number of positively charged residues(Arg+Lys) | 19（15.45%） |
| The instability index (II) | 94.69 |
| Grand average of hydropathicity (GRAVY) | -0.65 |
| Aliphatic index (AI) | 68.21 |
| Estimated half-life | 30 hours(mammalian reticulocytes, in vitro) |
|  | >20 hours(yeast, in vivo) |
|  | >10 hours(Escherichia coli, in vivo) |
