# Supplementary material for: Molecular cloning, characterization, and functional analysis of the uncharacterized C11orf96 gene
Source: BMC Vet Res. 2022 May 10;18:170. doi: 10.1186/s12917-022-03224-5 (PMC9086667; doi:10.1186/s12917-022-03224-5)
Supplement: Supplementary file 3 — Additional file 3: Table S2. The species of C11orf96 gene in this study. [file 12917_2022_3224_MOESM3_ESM.docx]

**Supplementary** **Table S2.** The species of C11orf96 gene in this study

| **Organism** | **Genbank ID** |
| --- | --- |
| ***Felis catus*** | **XM_006937308.4** |
| *Canis lupus dingo* | XM_025452536.2 |
| *Danio rerio* | NM_001386663.1 |
| *Panthera pardus* | XM_019470168.1 |
| *Ailuropoda melanoleuca* | XM_002929228.3 |
| *Camelus dromedarius* | XM_011000607.2 |
| *Xenopus tropicalis* | XM_002937301.5 |
| *Rattus norvegicus* | NM_001110055.2 |
| *Cygnus atratus* | XM_035552177.1 |
| *Mesocricetus auratus* | XM_005139339.3 |
| *Cavia porcellus* | XM_005001560.2 |
| *Macaca mulatta* | XM_015114423.2 |
| *Ovis aries* | XM_015101113.2 |
| *Bos taurus* | NM_001145035.1 |
| *Homo sapiens* | NM_001145033.2 |
| *Capra hircus* | XM_018059219.1 |
| *Mus musculus* | NM_001145034.1 |
| *Camelus ferus* | XM_032488510.1 |
| *Bubalus bubalis* | XM_025266197.1 |
| *Ochotona princeps* | XM_004585389.1 |
| *Gallus gallus* | NM_001145042.3 |
| *Oxyura jamaicensis* | XM_035329119.1 |
| *Sus scrofa* | XM_021085053.1 |
| *Cricetulus griseus* | XM_027422419.2 |
